# Effects of GABA_C_R and mGluR1 antagonists on contrast response functions of Sprague-Dawley and P23H rat retinal ganglion cells

**DOI:** 10.1371/journal.pone.0189980

**Published:** 2017-12-18

**Authors:** Ralph Jensen

**Affiliations:** Research Service, VA Boston Healthcare System, Boston, Massachusetts, United States of America; National Eye Centre, UNITED STATES

## Abstract

The GABA_C_R antagonist TPMPA and the mGluR1 antagonist JNJ16259685 have been shown previously to alter the sensitivity of retinal ganglion cells (RGCs) in the Sprague-Dawley (SD) rat and P23H rat (animal model of retinitis pigmentosa) to brief flashes of light. In order to better understand the effects of these antagonists on the visual responses of SD and P23H rat RGCs, I examined the responses of RGCs to a drifting sinusoidal grating of various contrasts. Multielectrode array recordings were made from RGCs to a drifting sinusoidal grating of a spatial frequency of 1 cycle/mm and a temporal frequency of 2 cycles/s. In both SD and P23H rat retinas, contrast response functions were found to have a variable shape across cells. Some cells showed saturation of responses at high contrast levels while others did not. Whereas 49% of SD rat RGCs exhibited response saturation, only 14% of P23H rat RGCs showed response saturation. TPMPA decreased the responses of saturating SD rat RGCs to low (6% to 13%) grating contrasts but increased the response to the highest contrast (83%) tested. JNJ16259685 did not significantly affect the contrast response functions of either saturating or non-saturating SD rat RGCs. In contrast, both TPMPA and JNJ16259685 increased the responses of saturating and non-saturating P23H rat RGCs to all grating contrasts. Neither TPMPA nor JNJ16259685 affected the contrast thresholds of SD rat RGCs, but both antagonists lowered the contrast thresholds of P23H rat RGCs. Overall, the findings show that GABA_C_R and mGluR1 antagonists have differential effects on the contrast response functions of SD and P23H rat RGCs. Notably, these receptor antagonists increase the responsiveness of P23H rat RGCs to both low and high contrast visual stimuli.

## Introduction

Contrast is an important parameter in assessing visual function. A person with reduced contrast sensitivity will have difficulty with many common daily tasks, such as detecting curbs or stairs, reading facial expressions, and driving at night. In clinical practice, contrast sensitivity charts are widely used to test the ability of a patient to perceive small differences in luminance between adjacent surfaces. In patients with retinal degenerative diseases, such as retinitis pigmentosa and age-related macular degeneration, contrast sensitivity may be diminished while visual acuity is still good as determined with a standard eye chart [1–5]. The neural mechanisms underlying the contrast sensitivity reduction are currently unknown.

In both retinitis pigmentosa and age-related macular degeneration, there is a loss of photoreceptors with concomitant remodeling of cells within the inner retina (for review see 6, 7). Details of the changes that emerge within the inner retina following degeneration of photoreceptors have come primarily from studies conducted in animal models of retinitis pigmentosa. Horizontal cells and bipolar cells, which are postsynaptic to photoreceptors, appear to be affected initially. Horizontal cells retract their dendrites [8, 9] and may grow processes directed towards in inner plexiform layer [10, 11]. Bipolar cells also retract their dendrites [8, 9], and in ON bipolar cells there is a down-regulation of dendritic mGluR6 receptors and TRPM1 channels [9, 11, 12]. Amacrine cells, which are postsynaptic to bipolar cells, are likewise affected. Morphological alterations in one type of amacrine cell–the AII amacrine cell–have been described in several animal models of retinitis pigmentosa [9, 13, 14]. In addition, these amacrine cells show elevated phosphorylation of the gap junction subunit Cx36 [15], which may increase electrical coupling between AII amacrine cells. In the inner retinas of degenerate retinas, alterations in the expression of AMPA, glycine, GABA_A_, GABA_C_ and NMDA receptors have been described [16, 17]. Increased levels of synaptic proteins in both bipolar cells and amacrine cells in the degenerate retina have also been reported [18], suggesting increased synaptic activity in these cells. These and very likely other, yet to be discovered, changes that take place in inner retinal neurons may contribute to the loss of contrast sensitivity in the patients with retinitis pigmentosa and age-related macular degeneration.

Previously, I showed that the GABA_C_R antagonist TPMPA and the mGluR1 antagonist JNJ16259685 increase the sensitivity of retinal ganglion cells (RGCs) in the P23H rat model of retinitis pigmentosa to brief flashes of light [19, 20]. The effects of these receptor antagonists are likely due to actions on cells in the inner retina since the receptors for these antagonists are found predominately on cell processes within the inner retina [21, 22]. In the interest of determining how TPMPA and JNJ16259685 may affect contrast sensitivity of RGCs, I have investigated the effects of these receptor antagonists on the responses of RGCs in P23H and SD rat retinas to a drifting sinusoidal grating of various contrasts.

## Materials and methods

### Animals

P23H-line 1 homozygous rats and Sprague-Dawley (SD) rats of 30–41 weeks of age were used in this study. Breeding pairs of P23H-line 1 homozygous rats were donated by Dr. Matthew LaVail (University of California, San Francisco). SD rats were obtained from Harlan Laboratories (Indianapolis, IN). The room light was kept on a 12 hr light/dark cycle using standard fluorescent lighting. During the light cycle, the illumination at the level of the cages was 100–200 lux. Both male and female animals were used.

This study was carried out in strict accordance with the recommendations in the Guide for the Care and Use of Laboratory Animals of the National Institutes of Health. The protocol was approved by the VA Boston Healthcare System Committee on Use and Care of Animals (Protocol Number: 304-J-060514). All surgery was performed in euthanized animals, and all efforts were made to minimize animal stress.

### Extracellular recordings

Following euthanasia of a rat with sodium pentobarbital (150 mg/kg, i.p.), an eye was removed and hemisected under room light. After removal of the vitreous, the eyecup was submerged in carboxygenated (95% O_2_, 5% CO_2_) Ames' Medium (supplemented with 2 g/L sodium bicarbonate and 1.5 g/L d-glucose). A square piece of retina measuring ∼2–3 mm on each side was cut out with Cohan-Vannas spring scissors (Fine Science Tools, Foster City, CA) and transferred with the ganglion cell side down onto a 64-channel planar Muse MEA (Axion Biosystems Inc., Atlanta, GA) with 30 μm-diameter nano-porous platinum electrodes at a 200 μm center-to-center spacing. To anchor the preparation, a piece of porous (30 μm pores) polycarbonate membrane (Sterlitech Corp., Kent, WA) was placed on the retina and this membrane was in turn held down by a nylon ring. To maintain viability of the retina, a gravity-flow system administered the carboxygenated Ames' Medium at a flow rate of 1.5 ml min^−1^. The temperature of the bath was maintained at 31 to 33°C with an in-line heater (Warner Instruments, Hamden, CT). The retina was superfused for at least 20 min before data acquisition to permit stabilization of spike amplitudes.

Raw data was digitized at 20 kHz and stored on a hard disk for offline analysis. Spike detection of single action potentials was performed using the Axion Biosystem software using a voltage threshold 5–6 fold the standard deviation of the noise over 200 Hz high-pass filtered traces. Principal component analysis of the spike waveforms was used for sorting spikes generated by individual cells (Offline Sorter, Plexon).

### Visual stimulation

Visual stimuli were generated with the PsychoPy (v1.81) package [23] and delivered to an overhead projector (Toshiba TDP-T420 DLP). The images from the projector were minified with external lenses, directed into the camera port of a Nikon microscope, and focused onto the photoreceptor surface of the retina with a 10X microscope objective.

Visual stimuli consisted of drifting sinusoidal gratings that were presented with a mean illuminance that equaled that of the background. The mean stimulus illuminance was adjusted by neutral density filters positioned adjacent to the projector output. The mean stimulus illuminance, measured with a digital lux meter (model 840020; Sper Scientific LTD, Scottsdale, AZ), was either 15 or 60 lux. (15 lux corresponds to 4.3 μW/cm^2^ as measured with an ILT900-R spectroradiometer from International Light Technologies.) Spatial frequency of the sinusoidal gratings was held constant at 1 cycle/mm, and temporal frequency was held constant at 2 cycles/s. All gratings were presented within a circular patch of 2.4 mm diameter, centered over the MEA. The neurons were tested with eight values of contrast (0, 4, 6, 8.5, 13, 26, 51, and 83%). Contrast was defined by the Michelson formula, 100% x (Lmax−L_min_)/ (L_max_ + L_min_), where L_max_ and L_min_ are the maximum and minimum illuminance levels of the sinusoidal grating. At each grating contrast, seven trials were presented. Each trial started with a 4 s presentation of a uniform field of the same mean illuminance as the grating. The drifting sinusoidal grating was then shown for 6 s. An interval of 20 s between trials was chosen to minimize possible effects of stimulation history.

### Drugs

The mGluR1 antagonist JNJ16259685 (Tocris Bioscience) and the GABA_C_R antagonist TPMPA (Tocris Bioscience) were added to the bath at 0.5 μM and 100 μM, respectively, using a calibrated syringe pump, as described previously [24]. Only one drug per retinal preparation was used to avoid possible long-term changes caused by the drug. The effects of a drug were examined only after the drug was bath applied for ~10 min to ensure stable responses.

### Data analysis

Sorted spikes from RGCs were imported into Neuroexplorer software (Nex Technologies) to create post-stimulus time histograms (PSTHs) with a 10 ms bin width, averaged across 7 repetitions of the same contrast. After discarding the first second at the beginning of each histogram (since cells often responded to the onset of the grating), each histogram was Fourier transformed with OriginPro10 software (OriginLab Corp.) to obtain the amplitude of the fundamental stimulus frequency (F1).

The response amplitude of each cell was obtained by subtracting the baseline (F1 amplitude) response determined with 0% grating contrast from the F1 amplitude obtained at each contrast level. The response amplitudes were used to construct a contrast response function, which was fitted with the hyperbolic ratio function [25] also known as the Hill equation

$$R=R_{max}\;x\;C^{n}/\left(C_{50}{}^{n}+C^{n}\right)$$

where R is response amplitude, R_max_ represents the maximum response amplitude, C is the stimulus contrast, C_50_ represents the contrast that produces R_max_/2, and n is a fitting exponent that determines the shape of the contrast response function.

Group comparisons of response amplitudes to various grating contrasts between drug-treated and control (pre-drug tested) were conducted with a two-tailed Student’s t-test. P values were corrected for multiple comparisons using the Holm-Bonferroni method. Holm-corrected P values < 0.05 were deemed significantly different. Medians are used to report contrast threshold data since for some cells the contrast threshold value was immeasurable (i.e., exceeded the highest contrast stimulus tested). Group comparisons of contrast thresholds were conducted with either the Wilcoxon signed-rank test or the Mann-Whitney U test, as appropriate. P values < 0.05 were considered statistically significant.

## Results

Behavioral experiments to evaluate contrast sensitivity in rats commonly present sinusoidal gratings of various contrasts [26–29]. Only one study [30] to my knowledge has reported on the performance of rat RGCs to a drifting sinusoidal grating that varied in contrast. I will therefore begin by describing the contrast response functions of RGCs in SD and P23H rat retinas before describing the effects of the GABA_C_R antagonist TPMPA and the mGluR1 antagonist JNJ16259685 on responses of the RGCs to the same grating stimuli.

### Contrast response functions of SD and P23H rat RGCs

Many SD and P23H rat RGCs were modulated by a full-field drifting sinusoidal grating (spatial frequency: 1 cycle/mm, temporal frequency: 2 cycles/s). However, 20 to 40% of recorded RGCs were unresponsive to the grating, even at high contrast; these cells were not included in the data analyses. Contrast response functions were obtained from 116 SD rat RGCs (9 retinas) and 69 P23H rat RGCs (9 retinas).

**Fig 1:**
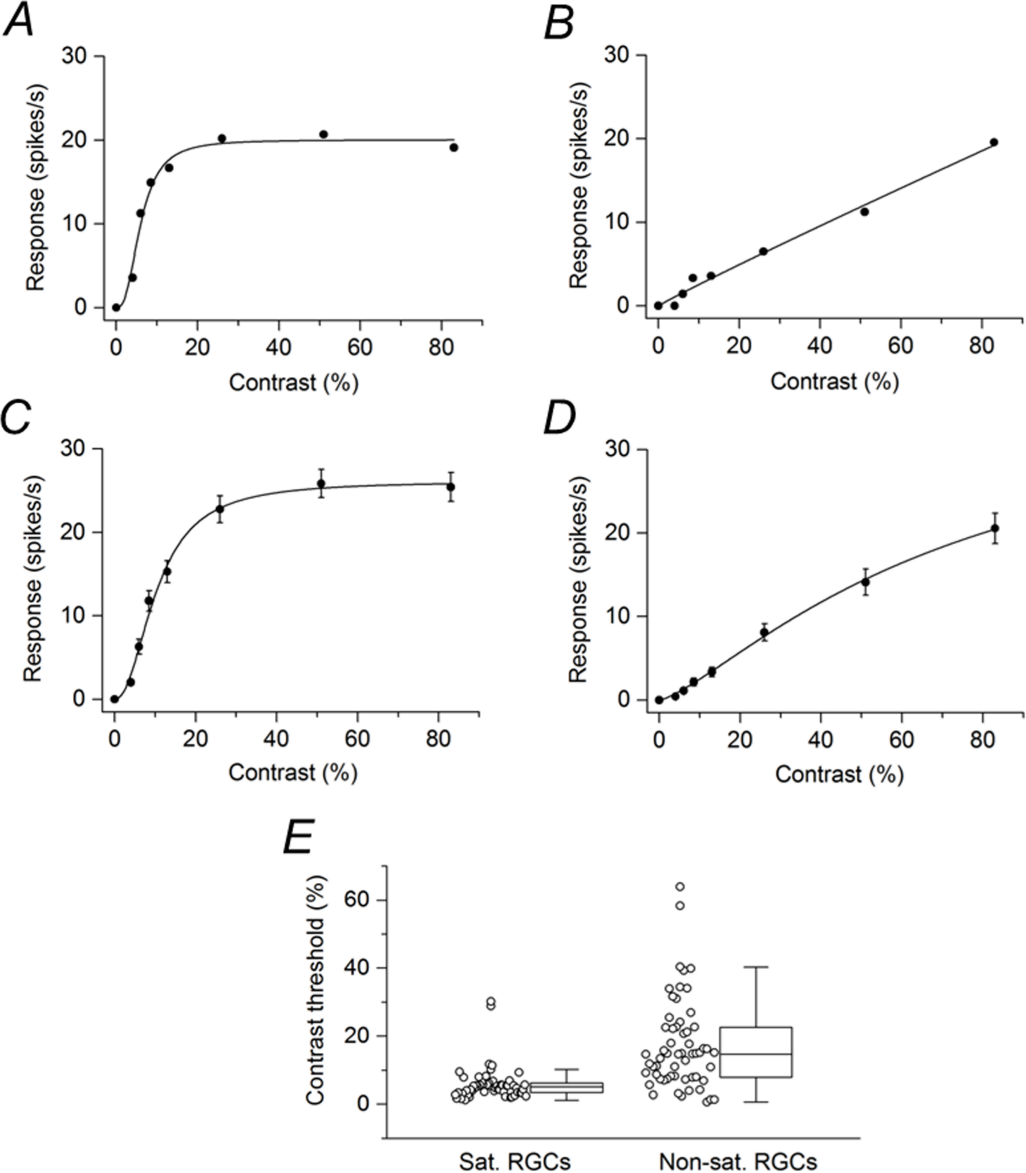
SD rat RGC responses to drifting sinusoidal grating (15 lux mean illuminance) of various contrasts. (A) Contrast response function from a representative cell that displayed response saturation. (B) Contrast response function from a representative cell that did not displayed response saturation. (C) Contrast response function averaged from saturating RGCs (n = 57). (D) Contrast response function averaged from non-saturating RGCs (n = 59). Data points in (C) and (D) are the mean ± SEM (errors smaller than the symbol size are not visible). (E) Contrast thresholds for saturating and non-saturating RGCs. Boxes represent the interquartile range (IQR) between first and third quartiles and the line inside represents the median. Whiskers denote the lowest and highest values within 1.5 x IQR from the first and third quartiles. Circles represent all data points.

For SD rat retinas, the mean illuminance of the grating, which varied in contrast (0, 4, 6, 8.5, 13, 26, 51, and 83%), was held constant at 15 lux. With increasing contrast, SD RGCs showed a monotonic increase in response amplitude. Many SD RGCs (n = 57) showed response saturation at high contrasts. This is illustrated for one SD RGC in Fig 1A. For this cell and other cells in this study, data were fitted with the hyperbolic ratio function (see Methods), which provided an excellent fit of the data as indicated by adjusted R^2^ values greater than 0.99. Many other SD RGCs (n = 59) did not show evidence of response saturation. This is illustrated for one SD RGC in Fig 1B. Based on the fit of the hyperbolic ratio function, RGCs were arbitrarily subdivided into two populations: saturating and non-saturating cells. Saturating RGCs included those cells whose value at 83% contrast was within 10% of the calculated plateau value; all other cells were categorized as non-saturating RGCs. Fig 1C shows the contrast response function averaged from the population of saturating RGCs, and Fig 1D shows the contrast response function averaged from the population of non-saturating RGCs. Saturating RGCs were very sensitive to changes in low contrast but not to changes in high contrast. Non-saturating RGCs on the other hand exhibited roughly a linear growth with increasing contrast. From the fitted hyperbolic ratio function, the contrast threshold of each cell could be determined. Contrast threshold was taken as a response amplitude of 2 spikes/s. The contrast threshold data are displayed as two box plots in Fig 1E. For the population of saturating SD rat RGCs, the median contrast threshold was 4.95%. For the population of non-saturating SD rat RGCs, the median contrast threshold was 14.8%. The difference between the medians was statistically significant (P < 0.001).

**Fig 2:**
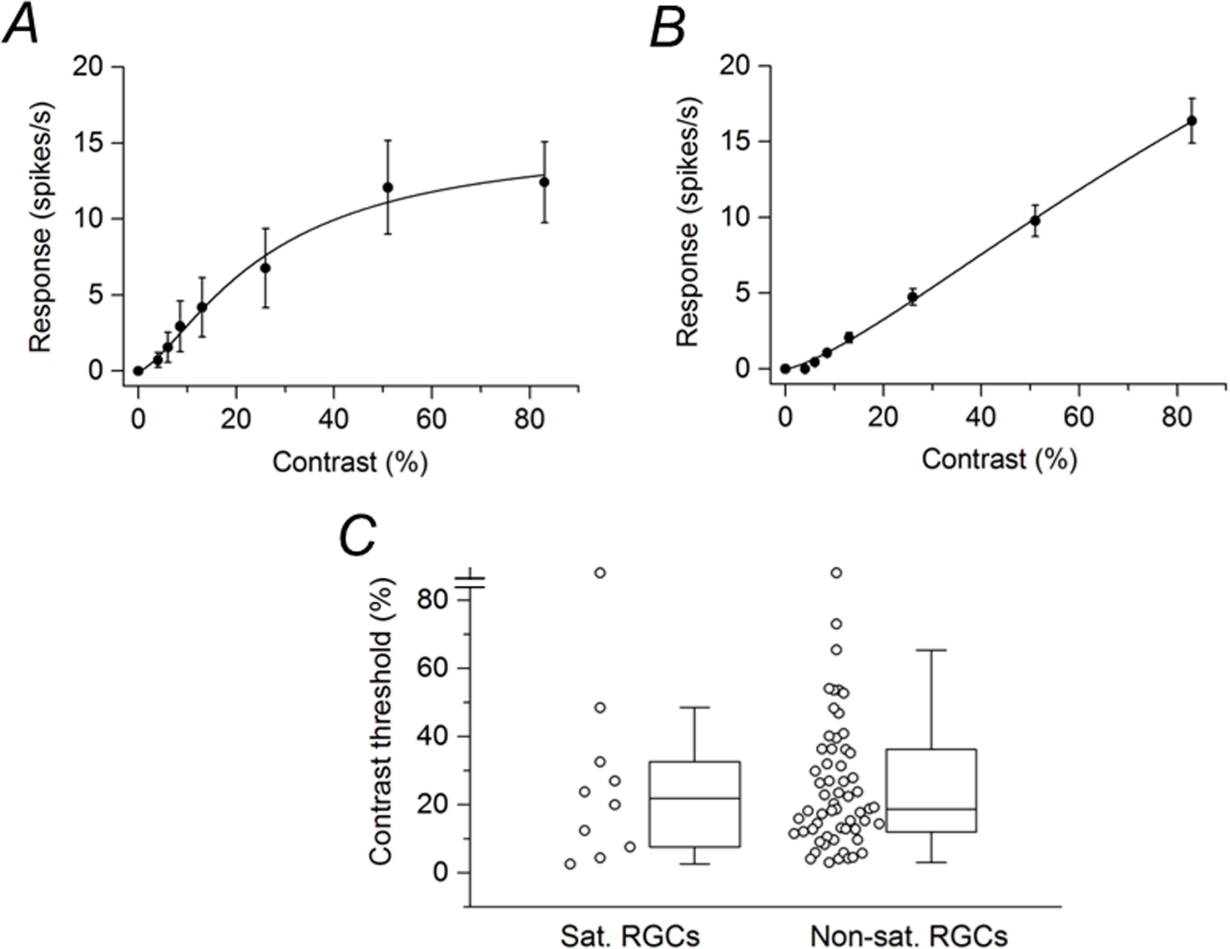
P23H rat RGC responses to drifting sinusoidal grating (60 lux mean illuminance) of various contrasts. (A) Contrast response function averaged from saturating RGCs (n = 10). (B) Contrast response function averaged from non-saturating RGCs (n = 59). Data points in (A) and (B) are the mean ± SEM. (C) Contrast thresholds for saturating and non-saturating RGCs. Boxes represent the interquartile range (IQR) between first and third quartiles and the line inside represents the median. Whiskers denote the lowest and highest values within 1.5 x IQR from the first and third quartiles. Circles represent all data points. Note the contrast threshold values for two cells (one data point in each box plot) were immeasurable (i.e., exceeded 83%).

In experiments with P23H rat retinas, data were collected with the mean illuminance of the grating set at 15 lux and at 60 lux. Many cells were not very responsive to the sinusoidal grating at 15 lux mean illuminance but gave robust responses at the mean stimulus illuminance of 60 lux. Even at this higher mean stimulus illuminance, some RGCs did not exhibit modulation of spike activity to the grating. Of 84 cells that did respond to the grating at this higher mean stimulus illuminance, 5 cells gave a response only to the highest contrast (83%) tested and 10 cells responded only to the two highest contrasts (51% and 83%). These cells were not included in the data analysis. Of the 69 P23H rat RGCs analyzed, only 10 cells showed response saturation. Fig 2A shows the contrast response function averaged from these saturating RGCs. Compared with saturating RGCs in the SD rat retina (Fig 1C), the P23H rat RGCs were less responsive to the drifting grating and less sensitive to changes in low contrast. Fig 2B shows the contrast response function averaged from the non-saturating RGCs (n = 59). The contrast response function was similar to that of non-saturating SD rat RGCs (Fig 1D). Contrast thresholds were determined for both saturating and non-saturating P23H rat RGCs. The data are displayed as two box plots in Fig 2C. For the population of saturating P23H rat RGCs, the median contrast threshold was 21.8%. For the population of non-saturating P23H rat RGCs, the median contrast threshold was 18.7%. The difference between the medians was not statistically significant (P = 0.878).

### Effects of GABA_C_R and mGluR1 antagonists on contrast response functions of SD rat RGCs

**Fig 3:**
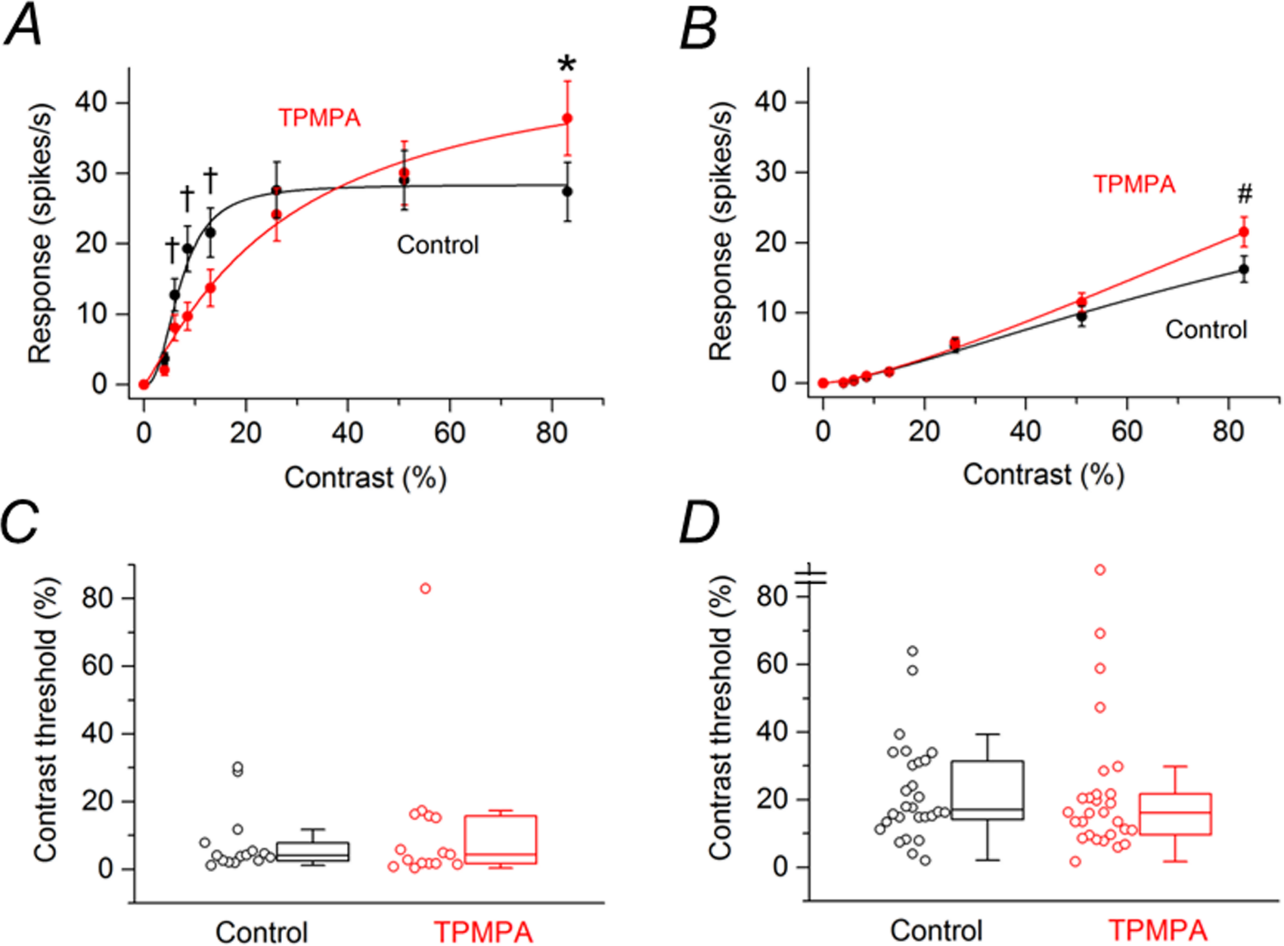
Effects of TPMPA on responses of SD rat RGCs to drifting sinusoidal grating (15 lux mean illuminance) of various contrasts. (A) Contrast response function from saturating RGCs (n = 15) before and after application of TPMPA. (D) Contrast response function from non-saturating RGCs (n = 28) before and after application of TPMPA. Data points in (A) and (B) are the mean ± SEM. * P < 0.05, # P < 0.01, † P < 0.001 (Holm-Bonferroni multiple correction). (C) Contrast thresholds for saturating RGCs before and after application of TPMPA. (D) Contrast thresholds for non-saturating RGCs before and after application of TPMPA. In (C) and (D), boxes represent the interquartile range (IQR) between first and third quartiles and the line inside represents the median. Whiskers denote the lowest and highest values within 1.5 x IQR from the first and third quartiles. Circles represent all data points. Note the contrast threshold value for one cell in (D) was immeasurable (i.e., exceeded 83%).

Of the 116 SD rat RGCs that were examined in the previous section, 43 cells (3 retinas) were treated with the GABA_C_R antagonist TPMPA and 38 cells (3 retinas) were treated with the mGluR1 antagonist JNJ16259685. Of the cells treated with TPMPA, 15 cells were saturating RGCs. Fig 3A shows the contrast response function averaged from these saturating RGCs before and after application of TPMPA. TPMPA significantly reduced the response amplitudes to contrasts ranging from 6 to 13% by 36–51% and increased the response amplitude to 83% contrast by 38%. Fig 3B shows the contrast response function averaged from non-saturating RGCs (n = 28) before and after application of TPMPA. TPMPA significantly increased the response amplitude by 33% to the highest contrast (83%) tested. Box plots in Fig 3C and 3D summarize the effects of TPMPA on the contrast thresholds of saturating and non-saturating SD rat RGCs, respectively. For the population of saturating SD rat RGCs, the median contrast thresholds were 4.01% before application of TPMPA and 4.36% after application of TPMPA to the retina. The difference between the medians was not statistically significant (P = 0.489). For the population of non-saturating SD rat RGCs, the median contrast thresholds were 17.0% before application of TPMPA and 16.1% after application of TPMPA to the retina. The difference between the medians was not statistically significant (P = 0.487).

**Fig 4:**
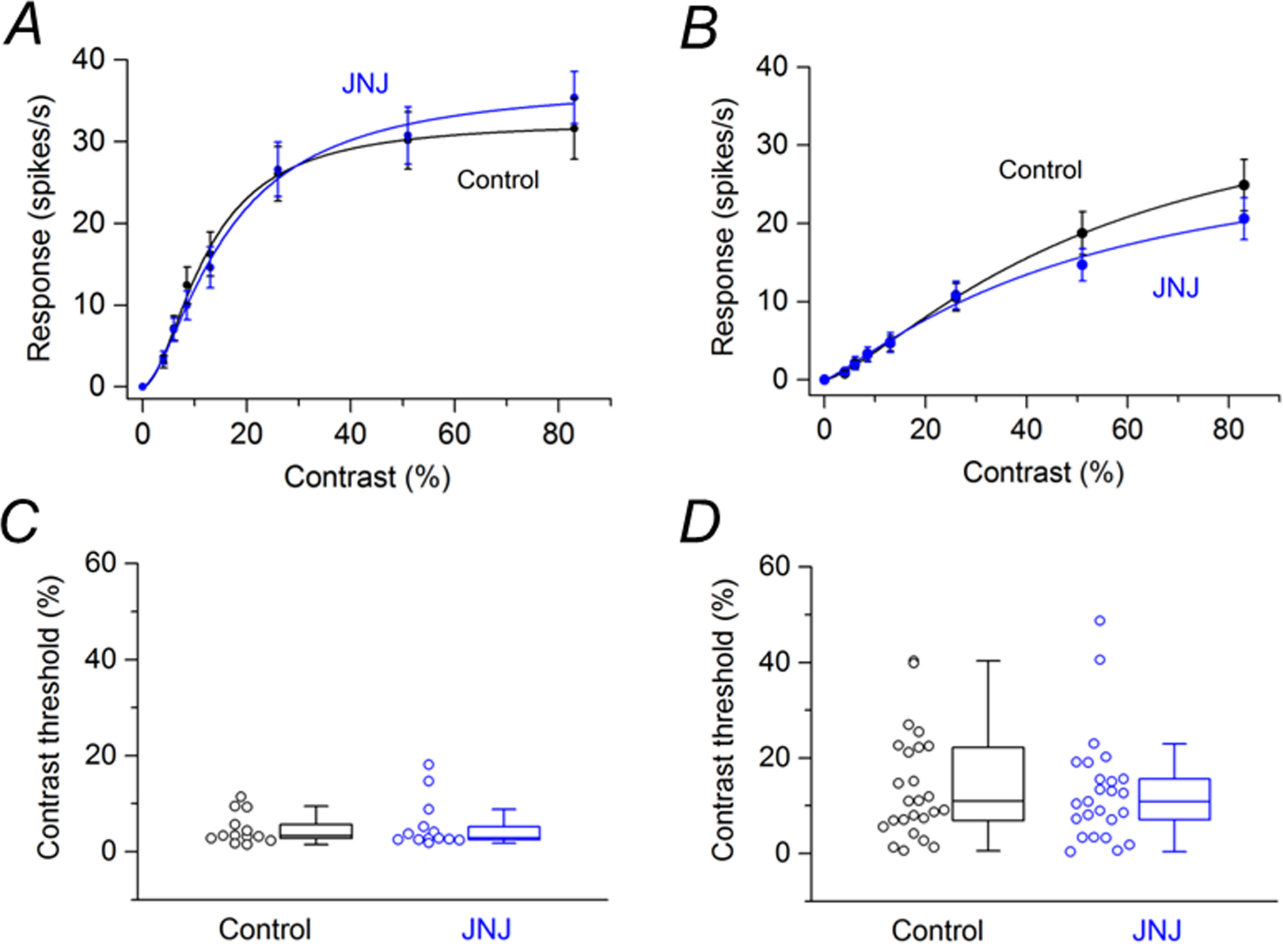
Effects of JNJ16259685 on responses of SD rat RGCs to drifting sinusoidal grating (15 lux mean illuminance) of various contrasts. (A) Contrast response function from saturating RGCs (n = 13) before and after application of JNJ16259685. (B) Contrast response function from non-saturating RGCs (n = 25) before and after application of JNJ16259685. (C) Contrast thresholds for saturating RGCs before and after application of JNJ16259685. (D) Contrast thresholds for non-saturating RGCs before and after application of JNJ16259685. In (C) and (D), boxes represent the interquartile range (IQR) between first and third quartiles and the line inside represents the median. Whiskers denote the lowest and highest values within 1.5 x IQR from the first and third quartiles. Circles represent all data points.

Of the cells treated with JNJ16259685, 13 cells were saturating RGCs. Fig 4A shows the contrast response function averaged from these saturating RGCs before and after application of JNJ16259685. JNJ16259685 had no statistically significant effect on the responses at any contrast. Of the cells treated with JNJ16259685, 25 cells were non-saturating RGCs. Fig 4A shows the contrast response function averaged from these non-saturating RGCs before and after application of JNJ16259685. JNJ16259685 had no statistically significant effect on the responses at any contrast. Box plots in Fig 4C and 4D summarize the effects of JNJ16259685 on the contrast thresholds of saturating and non-saturating SD rat RGCs, respectively. For the population of saturating SD rat RGCs, the median contrast thresholds were 3.36% before application of JNJ16259685 and 2.86% after application of JNJ16259685. The difference between the medians was not statistically significant (P = 0.100). For the population of non-saturating SD rat RGCs, the median contrast thresholds were 11.0% before application of JNJ16259685 and 10.9% after application of JNJ16259685. The difference between the medians was not statistically significant (P = 0.449).

### Effects of GABA_C_R and mGluR1 antagonists on contrast response functions of P23H rat RGCs

I previously found that both TPMPA and JNJ16259685 increase the sensitivity of P23H rat RGCs to brief flashes of light, shifting the intensity-response curves to the left [19, 20]. A leftward shift of the intensity-response curve is equivalent to removing a neutral density filter in front of the light source. I hypothesized that in the presence of either TPMPA or JNJ16259685 the responses of P23H rat RGCs to the drifting grating would be similar to that of increasing the mean illuminance of the grating (i.e., removing a neutral density filter in front of the light projector). I therefore tested the effects of TPMPA and JNJ16259685 on the responses of P23H rat RGCs with the mean illuminance of the grating set at 15 lux, which is the same mean illuminance that was used in examining the effects of TPMPA and JNJ16259685 on SD rat RGCs. Of the 84 P23H rat RGCs that were described previously, 35 cells (4 retinas) were treated with TPMPA and 49 cells (5 retinas) were treated with JNJ16259685. The 10 RGCs that were identified previously as saturating RGCs (based on the cells’ responses at 60 lux mean illuminance) were analyzed separately from the other cells, considering the finding that TPMPA had a differential effect on these cells in SD rat retinas (see Fig 3).

**Fig 5:**
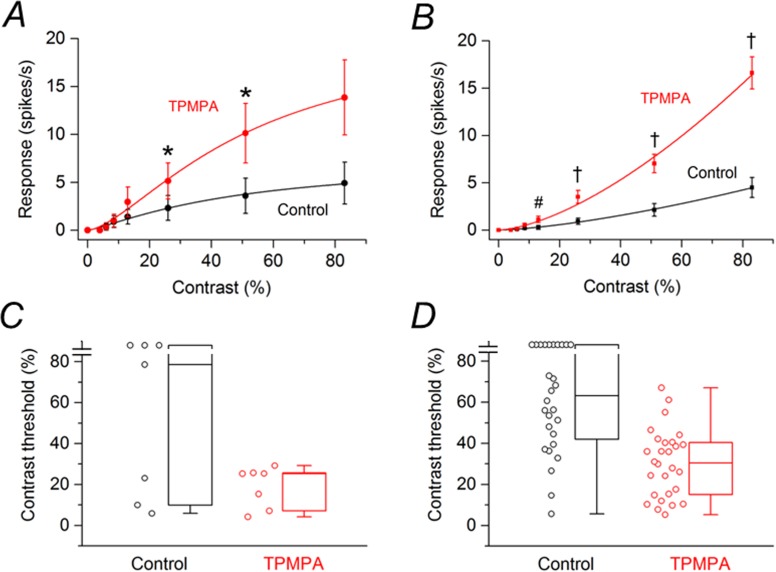
Effects of TPMPA on responses of P23H rat RGCs to drifting sinusoidal grating (15 lux mean illuminance) of various contrasts. (A) Contrast response function from saturating RGCs (n = 7) before and after application of TPMPA. (B) Contrast response function from non-saturating RGCs (n = 28) before and after application of TPMPA. * P < 0.05, # P < 0.01, † P < 0.001 (Holm-Bonferroni multiple correction). (C) Contrast thresholds for saturating RGCs before and after application of TPMPA. (D) Contrast thresholds for non-saturating RGCs before and after application of TPMPA. In (C) and (D), boxes represent the interquartile range (IQR) between first and third quartiles and the line inside represents the median, which in (C) in the presence of TPMPA is near the upper end of the box. Whiskers denote the lowest and highest values within 1.5 x IQR from the first and third quartiles. Circles represent all data points. Note the contrast threshold values for three cells in (C) and ten cells in (D) were immeasurable (i.e., exceeded 83%).

Of the 35 RGCs examined with TPMPA, 7 cells were saturating RGCs. Fig 5A shows the contrast response function averaged from these saturating RGCs before and after application of TPMPA. Before and after application of TPMPA, no response was observed from any cell at 4% contrast. At higher contrasts, the averaged response amplitudes increased by 13–182% in the presence of TPMPA. Statistically significant increases were obtained only with grating contrasts of 26% and 51%. Fig 5B shows the contrast response function averaged from non-saturating RGCs (n = 28) before and after application of TPMPA. Again, before and after application of TPMPA, no response was observed from any cell at 4% contrast. At higher contrasts, TPMPA increased the response amplitudes on average by 35–300%. Statistically significant effects were observed with contrasts from 13% to 83%. Box plots in Fig 5C and 5D summarize the effects of TPMPA on the contrast thresholds of saturating and non-saturating P23H rat RGCs, respectively. For the population of saturating P23H rat RGCs, the median contrast thresholds were 78.7% before application of TPMPA and 25.3% after application of TPMPA to the retina. The difference between the medians was statistically significant (P = 0.047). For the population of non-saturating P23H rat RGCs, the median contrast thresholds were 63.2% before application of TPMPA and 30.5% after application of TPMPA to the retina. The difference between the medians was statistically significant (P < 0.001).

**Fig 6:**
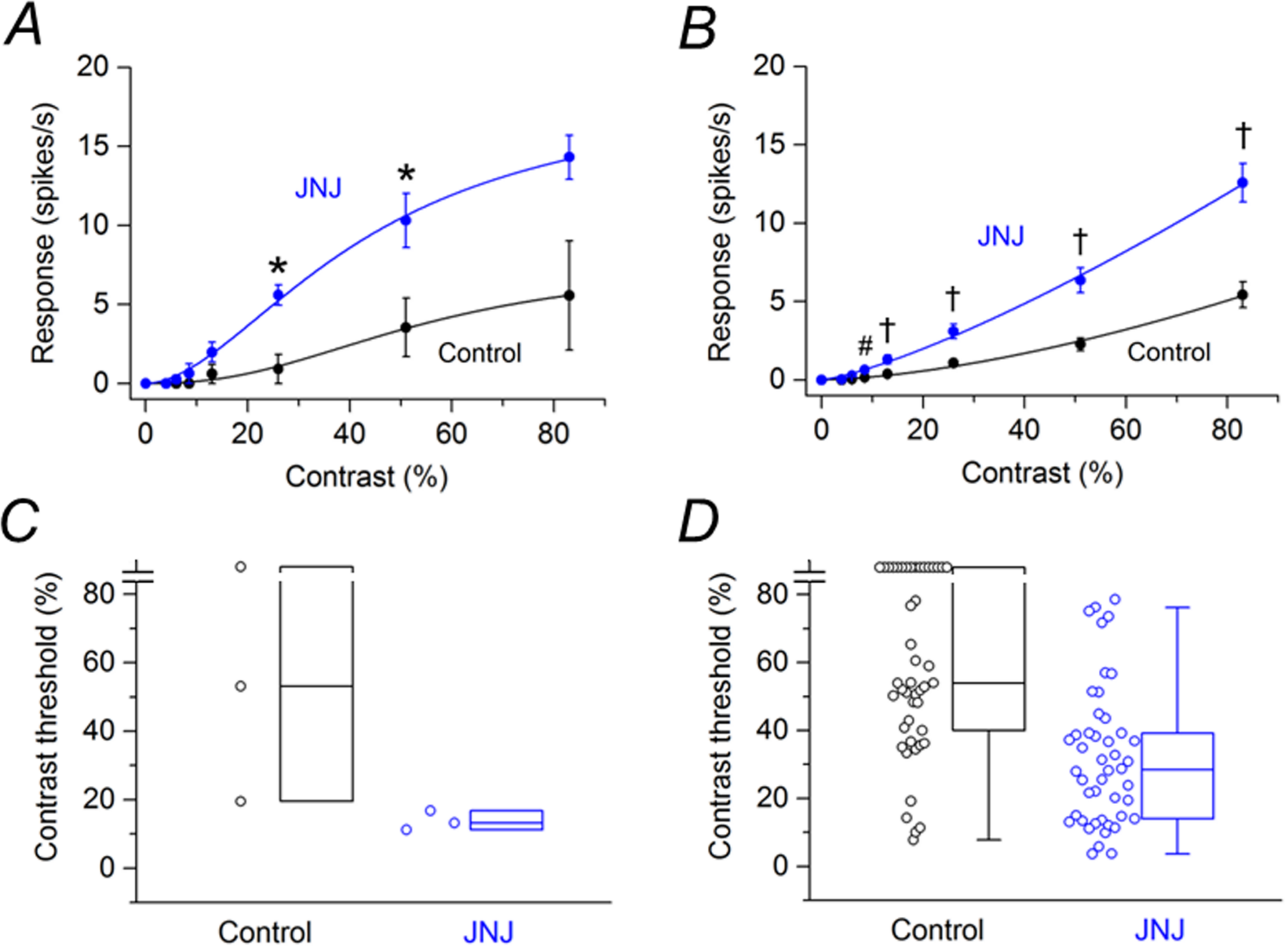
Effects of JNJ16259685 on responses of P23H rat RGCs to drifting sinusoidal grating (15 lux mean illuminance) of various contrasts. (A) Contrast response function from saturating RGCs (n = 3) before and after application of JNJ16259685. (B) Contrast response function from non-saturating RGCs (n = 46) before and after application of JNJ16259685. * P < 0.05, # P < 0.01, † P < 0.001 (Holm-Bonferroni multiple correction). (C) Contrast thresholds for saturating RGCs before and after application of JNJ16259685. (D) Contrast thresholds for non-saturating RGCs before and after application of JNJ16259685. In (C) and (D), boxes represent the interquartile range (IQR) between first and third quartiles and the line inside represents the median. Whiskers denote the lowest and highest values within 1.5 x IQR from the first and third quartiles. Circles represent all data points. Note the contrast threshold values for one cell in (C) and sixteen cells in (D) were immeasurable (i.e., exceeded 83%).

Of the 49 RGCs treated with JNJ16259685, only 3 cells were saturating RGCs. Fig 6A shows the contrast response function averaged from these saturating RGCs before and after application of JNJ16259685. Before and after application of JNJ16259685, no response was observed from any cell at 4% contrast. Before the application of JNJ16259685, no response was observed from any cell at either 6% or 8.5% contrast, and only one cell showed responses in the presence of JNJ16259685. At higher contrasts, JNJ16259685 increased the response amplitudes by 158–508%. Statistically significant increases were obtained with grating contrasts of 26% and 51%. Fig 6B shows the contrast response function averaged from non-saturating RGCs (n = 46) before and after application of JNJ16259685. At 4% contrast, no cell showed a response before addition of JNJ16259685 to the bathing solution and in the presence of JNJ16259685 only 1 cell elicited a response. At higher contrasts (6% to 83%), JNJ16259685 significantly increased the response amplitudes by 132–388%. Box plots in Fig 6C and 6D summarize the effects of JNJ16259685 on the contrast thresholds of saturating and non-saturating P23H rat RGCs, respectively. For the population of saturating P23H rat RGCs, the median contrast thresholds were 53.2% before application of JNJ16259685 and 13.2% after application of JNJ16259685 to the retina. However, the difference between the medians was found not to be statistically significant (P = 0.250). Clearly data on more cells are needed since this very small sample size (n = 3) has a reduced chance of detecting a true effect. For the population of non-saturating P23H rat RGCs, the median contrast thresholds were 54.0% before application of JNJ16259685 and 28.4% after application of JNJ16259685 to the retina. The difference between the medians was statistically significant (P < 0.001).

## Discussion

In this study I examined the effects of the GABA_C_R antagonist TPMPA and the mGluR1 antagonist JNJ16259685 on the responses of both SD and P23H rat RGCs to a drifting sinusoidal grating of various contrasts. Consistent with previous observations in the primate retina, some RGCs clearly show response saturation at high contrasts whereas others do not [31]. As in the primate retina, those RGCs in the SD rat retina that show response saturation are more sensitive to low contrast–the median contrast threshold of saturating SD rat RGCs is about 3-fold lower than the median contrast threshold of non-saturating SD rat RGCs. In behavioral investigations conducted on rats, Keller et al. [32] and Furtak et al. [33] found contrast thresholds to be 12–15%, whereas McGill et al. [27] and Douglas et al. [28] found contrast thresholds to be close to 5%. Differences in methodological approaches may explain the variation in contrast thresholds. In the present study, taking contrast threshold as response amplitude of 2 spikes/s, I found that the median contrast threshold of saturating SD rat RGCs is ~ 5%.

P23H rat RGCs respond poorly to the drifting sinusoidal grating at the mean stimulus illuminance (15 lux) that was used to collect data from SD rat RGCs. P23H rat RGCs did respond better when the mean stimulus illuminance was increased 4-fold to 60 lux. This finding is perhaps not surprising, given the loss of photoreceptors and diminished light sensitivity of remaining cone photoreceptors in these animals. Even at the higher mean illuminance, the median contrast threshold of saturating P23H rat RGCs is still (~4-fold) higher than that found for saturating SD rat RGCs. In fact, the median contrast thresholds of saturating and non-saturating P23H rat RGCs are similar (~ 20%). Interestingly, fewer saturating P23H rat RGCs were recorded in the present study. Whereas 49% of SD rat RGCs showed response saturation at high contrasts, only 14% of P23H rat RGCs showed response saturation at high contrasts. In the primate retina, Purpura et al. [34] reported that M cells (RGCs that project to the magnocellular layers of the lateral geniculate nucleus) show response saturation to a drifting sinusoidal grating whereas P cells (RGCs that project to the parvocellular layers of the lateral geniculate nucleus) do not. In the present study, I did not differentiate between M-like and P-like cells. Rats differ from primates in that the great majority of RGCs projects to the superior colliculus rather than the lateral geniculate nucleus [35–38]. Recent studies have shown that around 30 distinct types of RGC may exist in the rat retina [for review, see 39]. Which specific cell types exhibit response saturation will need to be determined in future experiments. It is noteworthy that Purpura et al. [34] reported that the shape of the contrast response curve of primate M cells is sensitive to the mean grating illuminance. At low mean stimulus luminance the contrast response function of M cells rises less steeply at low contrast (i.e., contrast gain is reduced) and does not show response saturation. It is therefore possible that more cells in the P23H rat retina would have shown response saturation if a higher mean grating illuminance had been used.

In P23H rats, I found that both JNJ16259685 and TPMPA increase the responses of saturating and non-saturating RGCs to all grating contrasts. Similar increases in responses are observed when the mean illuminance of the grating was increased from 15 lux to 60 lux. The effects of TPMPA and JNJ16259685 could be explained by an increase of the synaptic gain between (excitatory) bipolar cells and RGCs. GABA_C_ receptors, which are ligand-gated chloride channels, are found predominately on axon terminals of bipolar cells [22]. I previously hypothesized that in the degenerate retina there is an overstimulation of GABA_C_ receptors [19]. TPMPA would eliminate this GABA-mediated inhibition and thus the attenuation of light-evoked excitatory potentials in the axon terminals of bipolar cells. Previously, I reported that the effects of JNJ16259685 on the responses of P23H rat RGCs to flashes of light are similar to those of TPMPA [20]. I had postulated that the effects of JNJ16259685 may be mediated through a reduction in release of GABA onto GABA_C_ receptors. This mechanism would also explain the findings obtained with JNJ16259685 in the present study. In SD rats, I found that JNJ16259685 has no statistically significant effect on the contrast response function of RGCs. TPMPA also has no statistically significant effect on the contrast response function of non-saturating SD rat RGCs. The lack of effect of JNJ16259685 and TPMPA on contrast response functions could be explained by postulating that under my experimental conditions there is very little stimulation of GABA_C_ or mGlu1 receptors. However, I found that TPMPA decreases the responses of saturating SD rat RGCs to low (6% to 13%) grating contrasts and increases the response to the highest contrast (83%) tested. In the presence of TPMPA, the shape of the contrast response function begins to resemble that of non-saturating RGCs. It is unclear at the present time why blocking GABA_C_ receptors would preferentially affect saturating SD rat RGCs and decrease the responses of these cells to low contrast stimuli. Further studies will be needed to address this.

In conclusion, the results suggest that either a GABA_C_R antagonist or a mGluR1 antagonist may improve contrast sensitivity in patients with retinitis pigmentosa and possibly other retinal diseases in which there is photoreceptor degeneration with concomitant remodeling of cells within the inner retina.

## Supporting information

S1 Dataset This dataset contains the data points summarized in figures. Data for each figure are presented on separate sheets.
(XLSX)
